# Impaired p65 degradation by decreased chaperone-mediated autophagy activity facilitates epithelial-to-mesenchymal transition

**DOI:** 10.1038/oncsis.2017.85

**Published:** 2017-10-09

**Authors:** J Tang, M-N Zhan, Q-Q Yin, C-X Zhou, C-L Wang, L-L Wo, M He, G-Q Chen, Q Zhao

**Affiliations:** 1 Institute of Health Sciences, Shanghai Institutes for Biological Sciences, University of Chinese Academy of Sciences/Chinese Academy of Sciences and Shanghai Jiao Tong University School of Medicine (SJTU-SM), Shanghai, China; 2 Department of Pathophysiology, Key Laboratory of Cell Differentiation and Apoptosis of National Ministry of Education, Shanghai Jiao Tong University School of Medicine (SJTU-SM), Shanghai, China

## Abstract

Aberrant activation of nuclear factor-κB (NF-κB) has been observed in a wide range of human cancers and is thought to promote tumorigenesis and metastasis. As a central component of NF-κB pathway, p65 protein level is tightly regulated and could be subjected to proteasome degradation. Here we demonstrated that p65 can bind to HSC70 with four consensus recognition motif in its RHD domain and be constitutively transported to the lysosome membrane to bind with lysosome-associated membrane protein type 2A and degraded within the lysosome in two epithelial cell lines, proposing that p65 can be degraded by chaperone-mediated autophagy (CMA). Of great importance, there is a decreased CMA activity together with impaired degradation of p65 in a process of epithelial–mesenchymal transition (EMT). The resulted accumulation of p65 leads to higher NF-κB activity and contributes to the progression and maintenance of the EMT program. Taken together, our results define a novel regulatory mechanism for the important transcription factor p65, and these findings would shed new light on the inhibition of EMT, as well as metastasis of cancer cells.

## Introduction

The nuclear factor-κB (NF-κB) signaling is a major transducer of external signals, controlling the expression of a broad range of genes involved in cell survival, proliferation, stress response and inflammation. The mammalian NF-κB family consists of p50 (NF-κB1), p52 (NF-κB2), p65 (reLA), reLB and reLC, which all share an amino-terminal reL homology domain (rHD) and are regulated by eight IκB family members. Canonical activation of the NF-κB pathway depends on degradation of its inhibitor, IκBα, which retains the cytosolic distribution of p65/p50 heterodimer by direct binding with them.^1^ Hyper activation of NF-κB has been linked to abnormal proliferation, tumor initiation and progression in many types of cancer, including myeloma, leukemia and breast cancer.^2^

Epithelial–mesenchymal transition (EMT) is a process in which adherent epithelial cancer cells shed their epithelial traits and acquire mesenchymal fate, and it makes indispensable roles during embryonic development, wound healing and cancer metastasis.^3^ Cancer cells go through an EMT process to acquire enhanced potential for motility, enriched cancer stem cell portion and increased resistance to chemotherapy.^4, 5^ Complicated signaling pathways and proteins have been implicated in the regulation of EMT, including transforming growth factor β (TGFβ), tumor necrosis factor α (TNFα) signaling and transcription factors like Snail, Slug and Twist.^6^ Activation of NF-κB is believed to be required for the induction and maintenance of EMT through a series of mechanisms including regulation of Twist and Snail.^7, 8^

As the central component of NF-κB pathway, the protein level of p65 must be strictly regulated. However, the regulation mechanism of p65 at the post-translational level has not been fully elucidated although ubiquitin–proteasome degradation system and p62-mediated macroautophagy have been reported to regulate NF-κB activity through degradation of p65.^9, 10, 11^

Autophagy is the process that targets cellular components for degradation through lysosomes. Three types of autophagy have been described in mammalian cells, which are macroautophagy, microautophagy and chaperone-mediated autophagy (CMA).^12^ Cellular proteins containing a recognition motif chemically related to the pentapeptide Lys-Phe-Glu-Arg-Gln (KFERQ) could be recognized by cytoplasmic heat-shock chaperone HSC70 and its associated co-chaperones, being targeted to the lysosome membrane. After interacting with the lysosome-associated membrane protein type 2A (LAMP2A), the substrate is translocated through the membrane into the lysosome lumen with the assistance of luminal HSC70 chaperone and rapid degraded by luminal-resident proteases.^13, 14^

CMA has essential functions in maintenance of homeostasis, cellular quality control and recycling of amino acids for new proteins synthesis. Multifunction of CMA has been described owing to its selectively degradation of substrates, and dysfunction of CMA has been implicated in the pathogenesis of various diseases, such as neurodegenerative disease, metabolic disorders and lysosomal storage disorders.^15, 16, 17^ For example, CMA is upregulated in response to genotoxic insults to help maintain the genomic integrity and cell survival through regulated degradation of p-Chk1.^18^ In consistence with the divergent functions of macroautophagy, CMA exerts both pro-tumorigenesis and anti-tumorigenesis roles at different stage of cancer development.^19, 20^

Here in our work, we first demonstrate that p65 could be degraded by CMA as a novel classic substrate. We further established multiple EMT induction cell models and showed that p65 was accumulated in parallel with the decreased CMA activity, which represented higher NF-κB activity and contributed to EMT progression and maintenance.

## Results

### Degradation of p65 through lysosome

**Figure 1:**
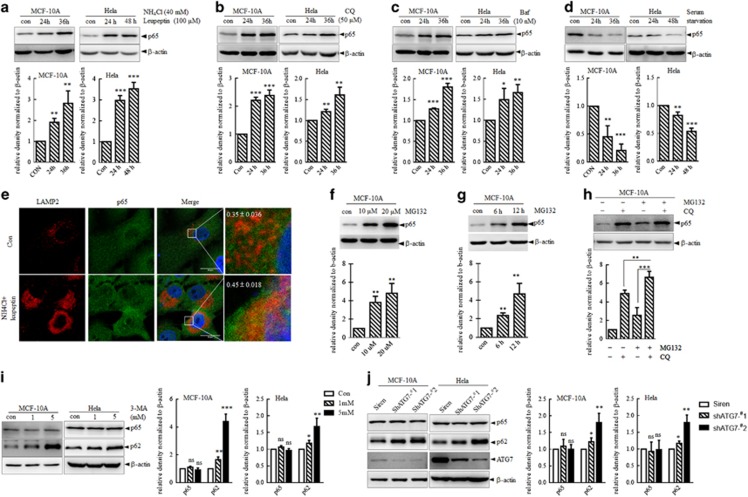
Degradation of p65 through lysosome. (**a**-**d**) MCF-10A and HeLa cells were treated with a combination of ammonium chloride (40 mM) and leupeptin (100 μM) (referred to NL hereinafter) (**a**), CQ (50 μM) (**b**) and Baf (10 nM) (**c**), or maintained with or without serum (**d**) for indicated time. Whole-protein lysates were subjected to immunoblots for indicated protein. Upper, at least three independent experiments were done, and representative image of immunoblot images were shown. Below, blot density was analyzed by ImageJ and quantification analysis was shown. ***P<0.001, **P<0.01 and *P<0.05 as compared with the con group. (**e**) Immunofluorescence co-staining of LAMP2 and p65 in MCF-10A cells treated with NL for 20 h or not. The colocalization between LAMP2 (red) and p65 (green) was analyzed by JAcoP plugin of ImageJ and values were presented as mean±s.e.m. Scale bar represents 20 μm. (**f**, **g**) **f**: MCF-10A cells were treated with MG132 for 12 h at indicated concentration, **g**: MCF-10A cells were treated with MG132 (10 μM) for indicated time. Whole-cell lysates were analyzed with immunoblots. Representative images were provided in left and quantification analysis of blot density was provided in right. *P<0.05 as compared with the con group. (**h**) MCF-10A cells were treated with MG132 (10 μM) or CQ (100 μM) alone or with a combination of MG132 and CQ and lysed for immunoblots for indicated proteins. Representative images were provided in left and blot density quantification analysis was shown in right. **P<0.01 and ***P<0.001 as compared with each group. (**i**, **j**) The effect of macroautophagy on the expression on P65. (**i**) MCF-10A and HeLa cells were treated with 3-MA for 24 h with indicated concentrations followed by immunoblotting for indicated proteins. (**j**) MCF-10A and HeLa cells were stably infected with Siren empty vector or shRNAs against ATG7 followed by immunoblots analysis of indicated proteins. Representative images were provided in left and quantification analysis of blot density of p65 and p62 were shown in right. ns, no significance. *P<0.05, **P<0.01 and ***P<0.001 as compared with con or siren in each group.

To uncover additional regulation mechanisms of the stability of p65, we investigated the role of lysosome in the degradation of p65 protein first by modulating lysosomal proteolysis with different treatments on both human non-tumorigenic immortalized breast epithelial cell line MCF-10A cells and cancer cell line HeLa cells. Incubation cells with a combination of ammonium chloride and leupeptin (referred to as NL hereinafter), which could block lysosomal hydrolyzes, increased the protein level of p65 in a time-dependent manner without affecting the mRNA level (Figure 1a and Supplementary Figure S1A). In addition, chloroquine (CQ) and Bafilomycin (Baf), inhibiting autophagy degradation in the lysosomes, could also induce the stabilization of p65 on the protein level without affecting the mRNA level in MCF-10A and HeLa cells (Figures 1b and c and Supplementary Figures S1B and S1C). Serum starvation activated autophagy activities and lysosome proteolysis, Figure 1d shows that serum starvation induced a time-dependent degradation of p65 protein in both MCF-10A and Hela cells without affecting the mRNA level (Supplementary Figure S1D). Furthermore, immunofluorescence co-staining of p65 and LAMP2, a known marker for lysosome, showed that p65 colocalize with lysosomes, and it was further enhanced when lysosomal degradation was inhibited by NL (Figure 1e). All the above results showed that p65 could be subjected to lysosome for degradation. In consistent with previous reports, treatment with MG132 induced an accumulation of p65 in a dose- and time-dependent manner in MCF-10A cells (Figures 1f and g). Strikingly, incubation with a combination of MG132 and CQ had a cumulative effect on the amount of p65 as compared with treated with MG132 or CQ alone (Figure 1h), indicating that degradation of p65 through lysosome was independent of proteasome.

To further determine whether macroautophagy contributes to p65 regulation, we analyzed p65 levels in MCF-10A and HeLa cells treated with 3-methyladenine (3-MA), a well-characterized and selective inhibitor of macroautophagy. As shown in Figures 1i, 3-MA induced the stabilization of p62, which was a well-recognized substrate of macroautophagy, without affecting the protein level of p65. Furthermore, macroautophagy activity was specifically inhibited by knocking down ATG7 with its two short hairpin RNAs (shRNAs). The results showed that in contrast to the stabilization of p62, p65 was unaffected upon macroautophagy inhibition by ATG7 knocking down (Figure 1j). Taken together, the results from pharmacological and genetic approaches indicated that p65 could be subjected to degradation through lysosome, which is a process independent of macroautophagy.

### p65 is a novel substrate of chaperone-mediated autophagy

**Figure 2:**
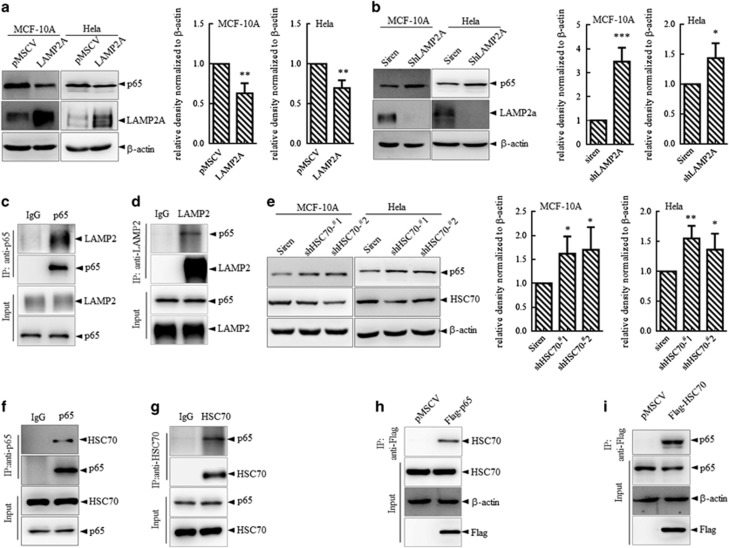
Regulation of p65 by chaperone-mediated autophagy. (**a**, **b**) MCF-10A and HeLa cells were stably infected with LAMP2A overexpression vector (**a**) or shRNA against LAMP2A (**b**) followed by immunoblots for indicated proteins. Left: representative images. Right: quantification analysis of the blot density of p65. *P<0.05, **P<0.01 and ***P<0.001 as compared with con. (**c**, **d**) Cell lysates from HEK293T cells were immunoprecipitated with anti-p65 (**c**) or anti-LAMP2 (**d**) antibodies. Precipitates were analyzed by western blots. (**e**) MCF-10A and HeLa cells were stably infected with two shRNAs against HSC70 followed by analysis of indicated proteins by immunoblots. Left: representative images. Right: quantification analysis of the blot density of p65. *P<0.05 and **P<0.01 as compared with the siren group. (**f**, **g**) Cell lysates from HEK293T cells were immunoprecipitated with anti-p65 (**f**) or anti-HSC70 (**g**) antibodies and precipitates were analyzed by western blots. (**h**, **i**) HEK293T cells were transfected with Flag-tagged p65 (**h**) or Flag-tagged HSC70 (**i**) and co-IP assay was performed with Flag M2 beads 48 h post transfection and followed by immunoblots for the indicated proteins.

As p65 degradation was independent of macroautophagy, we further tested whether p65 degradation was attributed to CMA. Substrate binding to and being translocated into lysosome by LAMP2A is the rate-limiting step for CMA, and cells use changes in LAMP2A levels to upregulate or downregulate CMA.^14^ So we first upregulated the CMA activity through stable overexpression of LAMP2A and found that the protein level of p65 was reduced, whereas the mRNA level remained unchanged (Figure 2a and Supplementary Figure S1E). Meanwhile, blockage of CMA activity through knocking down LAMP2A expression exerted the opposite effect on the p65 protein (Figure 2b and Supplementary Figure S1F). To further address the binding of LAMP2 with p65, we performed the co-immunoprecipitation assay at endogenous level and found that anti-p65 antibody significantly pulled down LAMP2 (Figure 2c) and p65 was also co-precipitated by anti-LAMP2 antibody (Figure 2d), suggesting an interaction between p65 and LAMP2.

HSC70 and its associated co-chaperones recognize cytosolic proteins with consensus motifs biochemically related to a pentapeptide KFERQ, delivering substrates to the lysosome membrane. We knocked down the HSC70 expression by two shRNAs and found that the reduced HSC70 upregulated the expression of p65 on the protein level without affecting its transcription (Figure 2e and Supplementary Figure S1G). Immunoprecipitation was also performed in HEK293T cells with antibodies against p65 (Figure 2f) or HSC70 (Figure 2g) followed by immunoblot analyzing, and the results indicated that p65 interacted with HSC70 at the endogenous level. Moreover, transfection of Flag-tagged p65 (Figure 2h) or Flag-tagged HSC70 (Figure 2i) in HEK293T cells, followed by co-IP with anti-Flag M2 beads further supported the interaction between HSC70 and p65. Collectively, we demonstrated that p65 could be recognized by HSC70 and bind with LAMP2. Meanwhile, HSC70 and LAMP2A could directly modulate the protein stability of p65. All the above data suggested that p65 was an autophagic substrate degraded through CMA.

### RHD domain of p65 carries HSC70 recognition motifs

The presence of one or several KFERQ-like motifs within the protein sequence is critical for a CMA substrate being recognized by HSC70 and subsequent degradation.^14^ To uncover the mechanism involved in p65 degradation through CMA, we began to explore the protein domains that involved in the interaction between HSC70 and p65.

**Figure 3:**
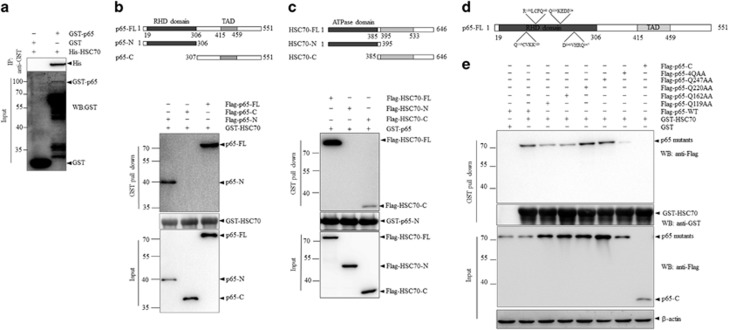
RHD domain of p65 carries HSC70 recognition motifs. (**a**) Bacterially expressed GST-p65 was incubated with His-HSC70, followed by pull down with GSH beads. The precipitates were detected for proteins as indicated. (**b**) Upper panel: schematic illustrations of p65 fragments. Lower panel: Flag-p65-full-length (FL), N-terminus (p65-N) or C-terminus (p65-C) was transfected into HEK293T cells. And cell lysates were incubated with recombinant GST-HSC70 followed by pull down with GSH beads. (**c**) Upper panel: schematic illustrations of HSC70 fragments. Lower panel: Flag-HSC70-FL/N/C was transfected into HEK293T cells. Cell lysates were incubated with recombinant GST-p65-N followed by GST-pull down and immunoblots analysis. (**d**) Identification for putative KFERQ motifs within the RHD domain of p65 protein sequence. (**e**) Flag-p65-C, Flag-p65-FL-WT and Flag-p65-mutants were expressed in HEK293T cells. Cells lysates were incubated with GST-HSC70 or GST alone, followed by GST-pull down and immunoblotting analysis as indicated.

First, glutathione S-transferase (GST)-pull down assay was performed, with recombinant GST-tagged p65 and His-tagged HSC70 being expressed in *Escherichia* coli and the results showed that despite the low expression efficiency of full-length GST-p65 owing to premature translation termination, GST-p65 but not GST significantly pull down His-HSC70, suggesting a direct interaction of p65 with HSC70 (Figure 3a).

To define which domain of p65 was involved in its binding with HSC70, as depicted in Figure 3b, Flag-tagged N-terminal, C-terminal, as well as full-length p65 were expressed in HEK293T cells, respectively, followed by incubation with recombinant GST-HSC70 and GSH beads. Immunoblotting analysis demonstrated that p65-FL and p65-N, but not p65-C could bind to GST-HSC70 (Figure 3b). To unravel which domain of HSC70 mediating its substrate recognition function, full-length, N-terminal and C terminal of Flag-tagged HSC70 were also expressed in HEK293T cells, respectively, as depicted in Figure 3c, followed by incubation with GST-p65-N and GSH beads. Immunoblots showed that C terminal of HSC70 was involved in its interaction with p65 (Figure 3c), which was in consistence with the previous reports that the 395–533 amino acids of HSC70 participated in its substrate recognition.^21^

On the basis of the indispensable role of RHD domain of p65 in its interaction with HSC70, we further analyzed the protein sequence of the RHD domain, searching for potential motifs chemically related to KFERQ.^14^ As depicted in Figure 3d, four putative CMA targeting motifs were identified within the RHD domain of p65. To figure out the functional recognition motifs, mutation strategies were utilized. Briefly, Gln (Q) and the adjacent amino acid within each motif were all mutated to alanine (AA), and single motif mutant or mutant of all the four motifs within p65 were constructed. The ability of Flag-tagged wild-type p65 or its mutants to be recognized by HSC70 was investigated by incubation with GST-HSC70 and GSH beads *in vitro*. As shown in Figure 3e, mutation of each motif could inhibit their interactions with HSC70 to some degree, whereas only mutation of all the four motifs could significantly block their interaction with HSC70, suggesting that all the four recognition motifs within p65 RHD domain contributed to its recognition by HSC70. Taken together, we demonstrated that p65 was a classic CMA substrate and defined four putative recognition motifs within its RHD domain.

### p65 protein accumulates in Snail-induced EMT process via impaired CMA activity

Aberrant activation of NF-κB has crucial roles in cancer development, working as a link between inflammation and tumor progression. Activation of NF-κB is believed to be required for the induction and maintenance of EMT through a series of mechanisms including regulation of EMT-related transcription factor Twist and Snail. To decipher the p65 regulation in EMT, we established multiple EMT induction cell model *in vitro*.

**Figure 4:**
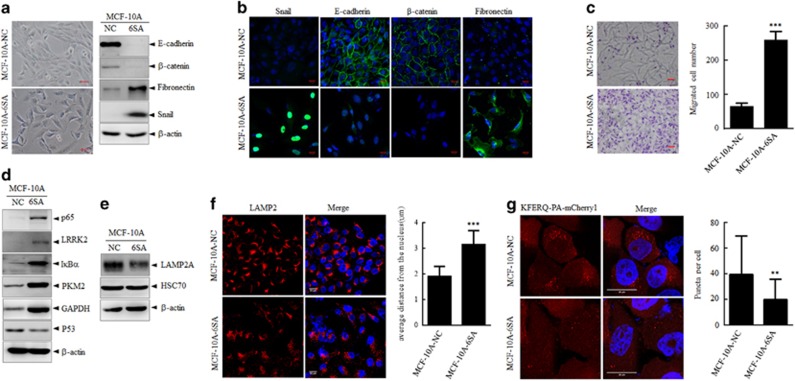
p65 protein accumulates in Snail-induced EMT process via impaired CMA activity. (**a**) MCF-10A cells were stably infected with lenti-NC and Snail-6SA, respectively, phase-contrast morphology observation (left) and expression of EMT marker proteins as indicated (right) were analyzed. Scale bar represents 20 μm. (**b**) Immunofluorescence staining of proteins as indicated together with re-staining of DAPI in MCF-10A-NC and MCF-10A-6SA cells. Scale bar represents 20 μm. (**c**) Transwell migration assay of MCF-10A-NC and MCF-10A-6SA cells. Left, representative images of migrated cells. Right, quantitative analysis of the migrated cell numbers in five different fields. Scale bar represents 20 μm. ***P<0.001 as compared with the migrated cell number of MCF-10A-NC cells. (**d**) Western blots of indicated proteins in MCF-10A-NC and MCF-10A-6SA cells. β-Actin served as internal control. LRRK2, IκBα, PKM2 and GAPDH were all reported CMA substrates, whereas wild-type p53 expressed in MCF-10A cells was utilized as a negative control. (**e**) Western blots of LAMP2A and HSC70 in MCF-10A-NC and MCF-10A-6SA cells. (**f**) Left: immunofluorescence staining of LAMP2 together with re-staining of DAPI in MCF-10A-NC and MCF-10A-6SA cells. Scale bar represents 20 μm. Right: the distance of LAMP2 from the nucleus in MCF-10A-NC and MCF-10A-6SA cells was measured using ImageJ. Twenty cells in each group were analyzed. Bar graphs represent the mean±s.d. The *P*-value was calculated using Student’s *t*-test. ***P<0.001 as compared with MCF-10A-NC group. (**g**) MCF-10A-NC and MCF-10A-6SA cells were stably infected with a KFERQ-PA-mCherry1 photoactivatable reporter, and after photoactivation by 405-nm light CMA was highly activated by serum starvation for 20 h. Left: representative images of KFERQ-PA-mCherry1 puncta number and distribution in MCF-10A-NC and MCF-10A-6SA cells. Right, quantitative analysis of the number of puncta per cell in >20 cells in at least eight different fields. Nuclei are labeled with DAPI. Scale bar represents 20 μm. **P<0.01 as compared with puncta number in MCF-10A-NC cells.

To begin with, snail-6SA, a stable variant of snail was utilized to induce EMT in MCF-10A cells. The cell morphological change and EMT marker alteration as detected through immunoblots and immunofluorescence indicated that snail-6SA induced a marked EMT programming in MCF-10A cells (Figures 4a and b). Enhanced cell mobility was also assessed through Transwell assay (Figure 4c). All these data supported that overexpression of Snail-6SA in MCF-10A cells successfully induced an EMT programming. In this process, the amount of p65 was markedly increased on the protein level, whereas the transcription level was unaffected (Figure 4d and Supplementary Figure S2), suggesting a decreased degradation of p65 in MCF-10A-6SA cells.

Considering the stability of p65 can be regulated by proteasome degradation and autophagy including CMA. We treated MCF-10A-NC and MCF-10A-6SA cells with chemical modulators of these degradation pathways and found that a comparable accumulation of p65 could be seen in either MCF-10A-NC or MCF-10A-6SA cells with proteasome inhibitor MG132 treatment (Supplementary Figure S3A). On the contrary, modulation of CMA activity in MCF-10A-NC cells resulted in the alteration of p65 protein, whereas this alteration could not be observed in MCF-10A-6SA cells, suggesting that p65 protein degradation through CMA pathway was inhibited in MCF-10A-6SA cells (Supplementary Figures S3B, S3C and S3D). In addition to the decreased degradation of p65 in MCF-10A-6SA cells, several well-recognized CMA substrates, including LRRK2, IκBα, PKM2 and GAPDH,^22, 23, 24, 25^ were also detected to be accumulated in MCF-10A-6SA cells (Figure 4d), indicating that decreased degradation of these CMA substrates might result from overall defect of the degradation system.

To investigate whether CMA activity was altered in this Snail-induced EMT process, we first detected the level of LAMP2A and HSC70, the two major effectors of CMA in MCF-10A-NC and 6SA cells, and the results showed that the LAMP2A protein was significantly decreased in MCF-10A-6SA cells compared with MCF-10A-NC cells, whereas HSC70 showed no alteration (Figure 4e). In addition, immunofluorescence analysis also showed that LAMP2 expression was decreased and exhibited a sporadic subcellular distribution in MCF-10A-6SA cells as shown by the increased average distance from the nucleus (Figure 4f), whereas the perinuclear distribution in MCF-10A-NC cells implies for a higher CMA activity.^26^

Most importantly, to detect specific CMA activity functionally, we conducted a photoactivated KFERQ-PA-mCherry1 reporter assay according to the protocol.^27^ Photoactivation of the KFERQ-PA-mCherry1 reporter allows detection of CMA activation as red fluorescent punctate patterns that result from redistribution of the fluorescent CMA substrate, KFERQ-PA-mCherry1, from the cytosol to the lysosomes membrane (Supplementary Figure S4). As indicated in Figure 4g, representative images and quantification analysis of the red fluorescent puncta within each cell showed that MCF-10A-6SA cells had less red fluorescent puncta compared with MCF-10A-NC cells, indicating a decreased CMA activity in MCF-10A-6SA cells.

Taken together, these data demonstrated a decreased CMA activity in MCF-10A-6SA cells, which leads to the accumulation of p65 protein during this Snail-induced EMT progress.

### P65 accumulation in parallel with CMA activity reduction in additional EMT models

**Figure 5:**
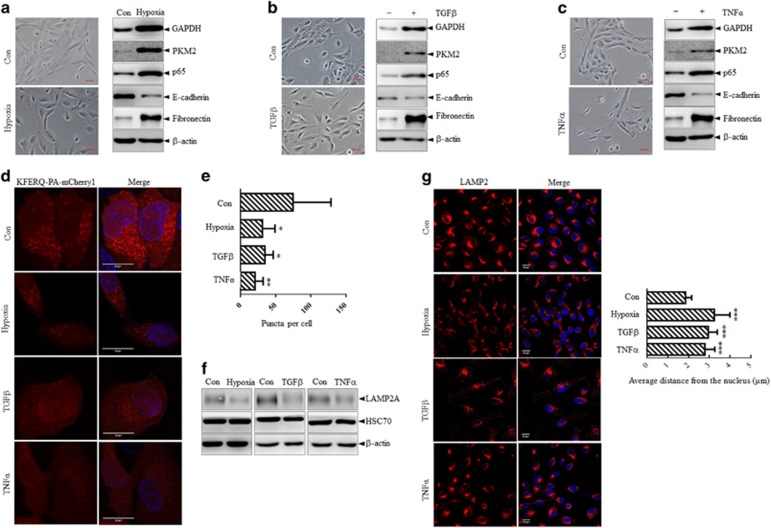
Reduction of CMA activity during hypoxia/TGFβ/TNFα induced EMT. (**a**–**c**) Induction of EMT in MCF-10A cells by hypoxia (**a**), TGFβ (**b**, 10 ng/ml) or TNFα (**c**, 10 ng/ml) treatment for 10 days. Phase-contrast morphology observation (left) and immunoblots for the indicated proteins (right) are shown. Scale bar represents 20 μm. (**d**, **e**) MCF-10A cells were stably infected with KFERQ-PA-mCherry1 and were stimulated with hypoxia/TGFβ/TNFα for EMT induction. Then, cells were photoactivated and maintained under serum starvation for 20 h to fully activate CMA. (**d**) Representative images of KFERQ- PA-mCherry1 puncta number and distribution in epithelial and mesenchymal MCF-10A cells induced by different treatments. Nuclei are labeled with DAPI. Scale bar represents 20 μm. (**e**) Quantitative analysis of the number of puncta per cell in >20 cells in at least eight different fields. *P<0.05 and **P<0.01 as compared with the puncta number in epithelial 10A-Con cells. (**f**) Immunoblots analysis of parental MCF-10A cells and MCF-10A cells induced by hypoxia, TGFβ and TNFα, respectively, for indicated proteins. (**g**) Left: immunofluorescence staining of LAMP2 together with re-staining of DAPI in parental MCF-10A cells and MCF-10A cells induced by hypoxia, TGFβ and TNFα, respectively. Scale bar represents 20 μm. Right: the distance of LAMP2 from the nucleus were measured using ImageJ. Twenty cells in each group were analyzed. Bar graphs represent the mean±s.d. The *P-*value was calculated using Student’s *t*-test. ***P<0.001 as compared with Con group.

To test whether the CMA activity changed during EMT induced by other factors, we exposed MCF-10A cells to TNFα, TGFβ stimulation or maintained them under hypoxia for several days to induce EMT. Immunoblotting and morphological images of MCF-10A cells under induction showed that hypoxia, TGFβ and TNFα could induce MCF-10A cells undergoing EMT efficiently (Figures 5a–c). The CMA substrates mentioned above were also detected by immunoblots and the results showed that GAPDH, PKM2, as well as p65 were all stabilized in the mesenchymal MCF-10A cells to an extent comparable to those in MCF-10A-6SA cells (Figures 5a–c). More importantly, photoactivation of the KFERQ-PA-mCherry1 reporter showed that the mesenchymal MCF-10A cells has less red fluorescent puncta, as indicated by the representative images and its quantification analysis (Figures 5d and e). In addition, a reduction on the protein expression of LAMP2A was detected in mesenchymal cells as compared with parental epithelial MCF-10A cells (Figure 5f). Immunofluorescence staining of LAMP2 was also conducted, in consistent with the phenomenon observed in MCF-10A-6SA cells, decreased intensity and sporadic distribution of LAMP2 were observed in the mesenchymal MCF-10A cells under different inductions (Figure 5g). All these results showed that CMA activity was inhibited during EMT progressing, either by transcription factor Snail overexpression or by hypoxia/cytokines induction, indicating that the compromised activity of CMA could be a general event during EMT process.

### Accumulation of p65 facilitates EMT progression

**Figure 6:**
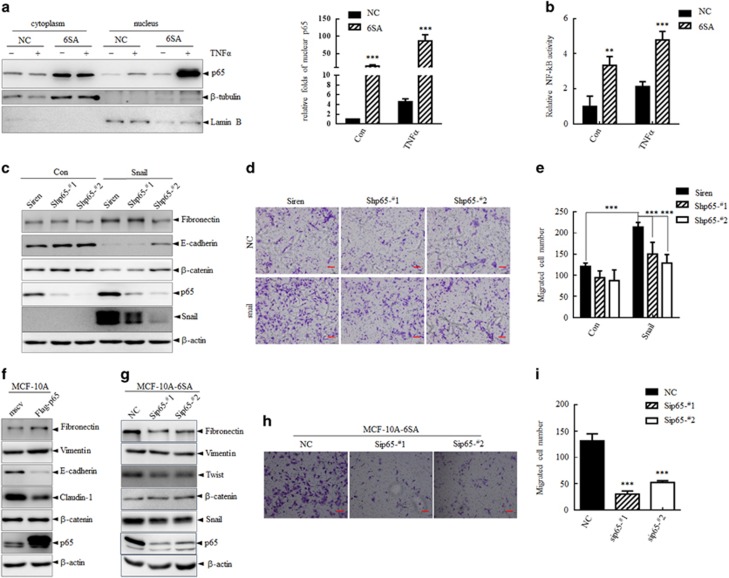
Accumulation of p65 facilitates EMT program. (**a**) MCF-10A-NC and MCF-10A-6SA cells were treated with TNFα (10 ng/ml) for 45 min followed by nuclear fractionation analysis with immunoblots. Lamin B and β-tubulin served as internal control for nucleus and cytoplasm, respectively (left). Right: quantification analysis of band density of nuclear p65 by ImageJ and normalized to that of lamin B. ***P<0.001 as compared with MCF-10A-NC cells in each group. (**b**) MCF-10A-NC and MCF-10A-6SA cells were transfected with luciferase reporter vector driven by six repeated κB reaction elements followed by TNFα stimulation and the transcriptional activity was determined by measuring the luciferase activity. **P<0.01 and ***P<0.001 as compared with MCF-10A-NC cells in each group. (**c**-**e**) MCF-10A cells were stably infected with Siren empty vector or two shRNAs targeting p65 followed by EMT induction with Snail overexpression. (**c**) Immunoblots for indicated proteins. (**d**) Cell migration ability were analyzed with Transwell assay. Representative images were shown in **d**, Scale bar represents 20 μm. (**e**) Quantitative analysis of the migrated cell numbers in five different fields. ***P<0.001. (**f**) MCF-10A cells were stably infected with pMSCV empty vector or Flag-p65 overexpression vector. Cell lysates were analyzed with indicated proteins by immunoblots. (**g**–**i**) 10A-6SA cells were transfected with siNC or siRNAs targeting p65. (**g**) Immunoblots analysis of indicated proteins. (**h**) Migration ability of siNC and sip65 cells were analyzed by Transwell assay. Representative images of migrated cells were shown in **h**, Scale bar represents 20 μm. (**i**) Quantitative analysis of the migrated cell numbers in five different fields. ***P<0.001 as compared with siNC cells.

Given that p65 accumulated in EMT process by reduced CMA activity, we next examined whether the stabilized p65 contributed to NF-κB activity in EMT induction. We assessed NF-κB transcriptional activity in MCF-10A-NC and MCF-10A-6SA cells both under basal level and TNFα stimulation. Consistent with a role as a transcription factor in nucleus, an increased fraction of p65 was found in the nucleus based on subcellular fractionation in MCF-10A-6SA cells compared with that in MCF-10A-NC cells, especially under TNFα treatment (Figure 6a). Furthermore, the higher NF-κB activity was also confirmed in MCF-10A-6SA cells compared with that in MCF-10A-NC cells through NF-κB luciferase reporter assay either in basal condition or with TNFα treatment (Figure 6b). Collectively, these results suggested that the stabilized p65 in MCF-10A-6SA cells resulted in higher NF-κB activity.

To further investigate whether this stabilization of p65 contributed to the EMT process, MCF-10A cells were stably infected with shRNAs specifically against p65 followed by overexpression of wide-type Snail. As expected, MCF-10A cells underwent EMT upon Snail overexpression as indicated by the reduction of E-cadherin and increase of fibronectin, which was partially reversed by knocking down of p65 (Figure 6c). More importantly, Snail overexpression could enhance cell mobility as analyzed by Transwell assay, which was significantly inhibited by knocking down of p65 (Figures 6d and e). In addition, immunoblotting analysis of the EMT markers showed that overexpression of p65 alone in MCF-10A cells partially induced EMT progress (Figure 6f). These results showed that p65 accumulation contributed to the induction of EMT.

It is of great importance for mesenchymal cells to maintain its mesenchymal properties after a successful EMT programming. To assess the involvement of accumulated p65 in EMT maintenance, we knocked down p65 in MCF-10A-6SA cells by specific siRNAs against p65, and assessed the mesenchymal properties through analyzing its EMT state and migration ability. The results showed that silencing of p65 partially reversed the epithelial–mesenchymal associated protein markers of MCF-10A-6SA cells and decreased cell migration ability, suggesting that elevated p65 also contributed to the maintenance of the mesenchymal properties (Figures 6g–i).

### Rescuing CMA activity through LAMP2a overexpression leads to compromised EMT

**Figure 7:**
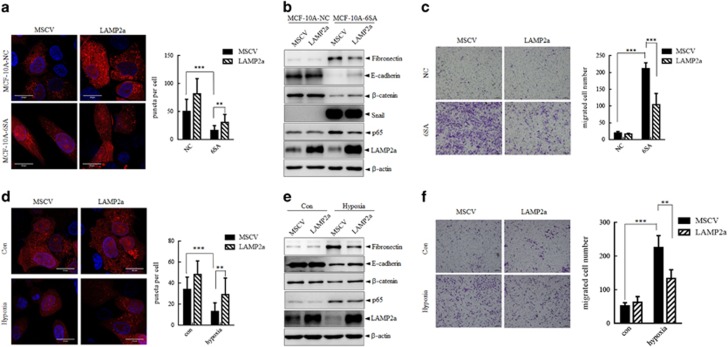
Rescuing CMA activity through LAMP2a overexpression leads to compromised EMT. (**a**–**c**) MCF-10A-NC and MCF-10A-6SA cells were infected with pMSCV or LAMP2a, respectively. (**a**) Left: representative images of KFERQ- PA-mCherry1 puncta number and distribution in each group. Nuclei are labeled with DAPI. Scale bar represents 20 μm. Right: quantitative analysis of the number of puncta per cell in >20 cells in at least eight different fields. **P<0.01 and ***P<0.001. (**b**) Immunoblots analysis of indicated proteins. (**c**) Migration ability were analyzed by Transwell assay. Representative images of migrated cells were shown left and quantitative analysis of the migrated cell number in five different fields were shown right. Scale bar represents 20 μm. ***P<0.001. (**d**–**f**) MCF-10A cells were stably infected with pMSCV or LAMP2a, respectively, and followed by incubation under hypoxia for EMT induction. (**d**) Left: representative images of KFERQ- PA-mCherry1 puncta number and distribution in each group. Nuclei are labeled with DAPI. Scale bar represents 20 μm. Right: quantitative analysis of the number of puncta per cell in >20 cells in at least eight different fields. **P<0.01 and ***P<0.001. (**e**) Immunoblots analysis of indicated proteins. (**f**) Migration ability was analyzed by Transwell assay. Representative images of migrated cells were shown left and quantitative analysis of the migrated cell number in five different fields was shown right. Scale bar represents 20 μm. **P<0.01 and ***P<0.001.

Given that the compromised p65 degradation contributed to the EMT process, as well as the maintenance of the mesenchymal properties. We further investigated whether this compromised p65 degradation and elevated EMT could be reversed by increased CMA. First, we overexpressed LAMP2a in MCF-10A-NC and MCF-10A-6SA cells. The photoactivation of the KFERQ-PA-mCherry1 reporter showed that the overexpression of LAMP2a in both cells had more red fluorescent puncta, as indicated by the representative images and their quantification analysis (Figure 7a), which indicated the increased CMA activity in both cells. Along with the increased CMA activity, the protein level of p65 were partially decreased, meanwhile the protein level of EMT markers showed that the overexpression of LAMP2a partially reversed the mesenchymal properties (Figure 7b). In addition, the increased cell mobility induced by the Snail overexpression could be partially rescued by the overexpression of LAMP2a as analyzed by Transwell assay (Figure 7c). Furthermore, the MCF-10A cells were stably infected with pMSCV or LAMP2a, respectively, and followed by incubation under hypoxia for EMT induction. The photoactivation of the KFERQ-PA-mCherry1 reporter showed that hypoxia could impair the CMA activity, as indicated before, whereas the overexpression of LAMP2a could rescue the CMA activity to some extent (Figure 7d). Meanwhile, immunoblotting analysis of the EMT markers showed that the overexpression of LAMP2a could partially reverse the EMT induced by hypoxia (Figure 7e). In addition, the increased cell mobility in EMT induced by hypoxia could also be partially rescued by the overexpression of LAMP2a as analyzed by Transwell assay (Figure 7f). The above results indicated that the compromised p65 degradation and elevated EMT could be partially rescued by increased CMA through LAMP2a overexpression.

In summary, we demonstrate for the first time that p65 was a novel substrate of CMA. Moreover, the accumulation of p65 because of decreased CMA activity during EMT leads to higher NF-κB activity and contribute to the progression and maintenance of the EMT program.

## Discussion

As a transducer of external signals to internal responses, NF-κB could be activated by bacterial and viral antigens, ultraviolet irradiation, stress and cytokines and has a key role in cellular homeostasis and immunoregulation. P65, the central component of NF-κB pathway, could be subjected to ubiquitin modification and subsequent proteasome degradation. Nuclear E3 ligase PDLIM2 and ECS E3 ligase complex are reported E3 ligases that target p65 for proteasome degradation, contributing to the termination of NF-κB activity timely and properly.^9, 10^ PDLIM2-mediated ubiquitination of p65 shuts down NF-κB activity either by targeting p65 for proteasome degradation in the cytosol or transporting p65 to specific nuclear compartments known as PML bodies.^10^ PDLIM2-deficient mice exhibit uncontrolled inflammation. There are other evidence that conditional medium from cancer cell line induced the M2 differentiation of BMDM through selective degradation of p65 by p62-dependent macroautophagy.^11^ In our study, we provided evidences that p65 was subjected to CMA degradation independent of its proteasome degradation, whereas hindered degradation of p65 through CMA lead to higher NF-κB activity during EMT process. We also provided evidences that macroautophagy did not contribute to its lysosomal degradation under basal level. Nevertheless, we cannot rule out the possibility that further modifications such as acetylation and phosphorylation could regulate p65 degradation through CMA.

Autophagy has been reported to have divergent roles in different stage of cancer development in different tissues. As for the functional relevance of macroautophagy and CMA, the dual role of macroautophagy in tumor development may shed light on the promising function of CMA. On one side, CMA activity is reported to be elevated in cancer cell lines and tumor tissues compared with normal cell and normal tissue. Inhibition of CMA activity by LAMP2A knocking down inhibits the tumor growth and metastasis *in vitro* and *in vivo,* suggesting a tumor-promoting role of CMA.^20, 28^ Besides, several proteins contribute to inhibition of cell proliferation and cancer metastasis have been reported to be degraded through CMA, including PED and RND3.^29, 30^ On the contrary, CMA has been proved to have regulatory functions in degradation of proteins that could promote the oncogenesis and tumor development of cancer, such as HIF-1α and PKM2.^21, 25^ For instance, impaired degradation of PKM2 through CMA could result in tumor growth and metastasis ability promoting *in vitro* and *in vivo*. Here we have demonstrated that CMA activity was inhibited during EMT, resulting in the stabilization of a series of its substrates, including PKM2, GAPDH and p65, which are all of potential to promote the survival and metastasis of cancer cells, suggesting an inhibitory role of CMA during cancer metastasis at least in the process of EMT.^31, 32^

EMT is a vital process of embryonic development, tissue remodeling, wound healing and even cancer metastasis. Massive efforts have been made to try to clarify factors and signaling that make contributions to EMT process. EMT can be induced by various transcription factors (that is, Twist, Snail, Slug, ZEB1 and ZEB2), hypoxia microenvironment, as well as a variety of cytokines and growth factors such as TGFβ and TNFα. NF-κB is reported to be essential for the induction and maintenance of EMT through a series of mechanisms. Evidences showed that activated NF-κB could stabilize Snail in a CSN2-dependent manner and also transcriptionally upregulate transcriptional factor Twist.^7, 8^ In our work, we proved that p65 stability is improved during EMT induced by either Snail overexpression or cytokines stimulation, leading to higher NF-κB activities and contributing to EMT induction and maintenance. Thus, it is likely that there is a positive feedback loop between EMT and NF-κB signaling, that is, EMT induction could stabilize p65 and lead to higher NF-κB activity, whereas higher NF-κB activity further promote the EMT program by stabilization of Snail and transcriptional activation of Twist. This may explain why silencing of p65 significantly decreased the protein level of Snail as depicted in Figure 6c. We suppose that inhibiting the stabilization of p65 through CMA or promoting the degradation of p65 could be of great potential to the blockage of EMT program.

In summary, we have delineated a novel pathway for p65 degradation that is independent of the proteasome. We demonstrated that p65 interacted at endogenous levels with HSC70 and LAMP2A, which were key CMA effectors and was depredated through the CMA pathway in MCF-10A and HeLa cells. In addition, we have found an obvious accumulation of p65 because of decreased CMA activity during EMT induced by Snail overexpression, hypoxia treatment, as well as TGFβ or TNFα stimulation. Moreover, we proved that the p65 accumulation leads to higher NF-κB activity and facilitated the induction and maintenance of EMT.

## Materials and methods

### Cell lines and reagent

MCF-10A cells were obtained from American type culture collection (Manassas, VA, USA) and authenticated by BIOWINGS (Shanghai, China) using the short tandem repeat profiling method. MCF-10A cells were maintained in Dulbecco’s modified Eagle’s medium/F12 medium supplemented with 5% horse serum, 10 μg/ml insulin, 20 ng/ml EGF, 0.5 μg/ml hydrocortisone and 100 ng/ml cholera toxin. HeLa and HEK293T cells were obtained from the cell bank of Chinese Academy of Science, and maintained in Dulbecco’s modified Eagle’s medium supplemented with 10% fetal bovine serum. All cells were cultured in a humidified atmosphere of 5% CO_2_ and 95% air at 37 °C. For hypoxia culture, 1% O_2_ was generated by flushing a 94% N_2_/5% CO_2_ mixture into the incubator (Thermo, Waltham, MA, USA).

Cell transfection was performed using Lipofectamine 2000 according to the manufacturer’s instruction, and shRNA targeting sequences against indicated genes were listed in Supplementary Table 1.

### Immunoblots

Detailed procedure was described elsewhere.^33^ All antibodies used were listed in Supplementary Table 4.

### Reverse transcriptase–PCR and quantitative real-time PCR

Detailed procedure of total RNA isolation and quantitative reverse transcriptase–PCR were described elsewhere.^33^ Paired primers for indicated genes were listed in Supplementary Table 2.

### PCR site-directed mutagenesis

Recognition motifs biochemically related to KFERQ were identified as stated in Dice *et al.*^14^ and four KFERQ motifs were identified within p65 protein sequence. To clarify the recognition motifs for HSC70, single motif mutation and mutation of all the four motifs were constructed. The mutation strategy was that Q and the adjacent amino acid were mutated to AA. PCR site-directed mutagenesis kit from Vazyme (Nanjing, China) were used for the mutation and the mutagenic oligonucleotides designed to produce the desired point mutations were listed in Supplementary Table 3.

### Fluorescence microscopy

Coverslip grown cells were fixed in 4% paraformaldehyde and permeabilized in methanol for 10 min. After blocking in 2% bovine serum albumin for 1 h, coverslips were incubated with diluted primary antibodies overnight at 4 °C. Coverslips were subsequently washed three times. Secondary antibodies (Alexa Fluor secondary 488, 595; Invitrogen, Carlsbad, CA, USA) were applied at 1:200 dilution for 1 h at room temperature. Before mounting with vectorshield with 4,6-diamidino-2-phenylindole (DAPI), coverslips were washed three times more in phosphate-buffered saline. Immunofluorescence pictures were taken with the Nikon Eclipse Ti (Nikon, Kanagawa, Japan). Images were analyzed using Image J (National Institutes of Health, Bethesda, MD, USA) software, and colocalization was analyzed by JAcoP Plugin with the overlay coefficient value taken as the indication for their colocalization. The distance of LAMP2 from the nucleus was taken as the index for LAMP2 localization.^34^ The distance of LAMP2 from the nucleus was measured using ImageJ with the analyzing particle function.

### KFERQ-PA-mCherry1 reporter assay

The original pPA-mCherry1-N1 was a gift from Vladislav Verkhusha (Addgene plasmid # 31928, Cambridge, MA, USA).^35^ The MCS and the PA-mCherry1 sequence of pPA-mCherry1-N1 was cloned and inserted into the pLVX-IRES-puro vector. The pLVX-KFERQ-PA-mCherryN1 expression plasmid was constructed as reported in Koga *et al.*^27^ MCF-10A cells were stably infected with pLVX-PA-mCherryN1 and pLVX-KFERQ-PA-mCherryN1 lentivirus. For CMA activity analysis, MCF-10A cells under stimulation or not were photoactivated by a 405 nm light emitting laser, followed by serum starvation for 20 h or not. Cells were fixed and co-stained with DAPI. The fluorescence pattern of the reporter was analyzed by fluorescence microscopy. Images were acquired with Nikon Eclipse Ti (Nikon). Activation of CMA activity was calculated as the number of fluorescence puncta per cell and quantification analysis was performed with ImageJ software.

### Recombinant expression and GST-pull down

Recombinant GST-tagged protein were expressed in *E*. coli by induction with 0.2 mM IPTG for 12 h at 22 °C. GST alone and GST-tagged fusion proteins were incubated with Glutathione Sepharase 4B overnight at 4 °C. After washing, the beads for three times GSH beads were re-incubated with His-tagged proteins or whole-cell lysate from HEK293T cells expressing Flag-tagged proteins for 6 h at 4 °C. Then, the precipitations were washed five times and eluted by the sodium dodecyl sulfate sample buffer, and the supernatant elution was analyzed by western blot.

### Migration ability assay

Cell migration ability was determined by Corning (Lowell, MA, USA) transwell insert chambers, and the detailed procedure was presented elsewhere.^36^

### Statistical analyses

All experiments were repeated at least three times or otherwise stated. No statistical methods were used for sample size selection. Results were blindly obtained and analyzed. The estimate of variation within each group was similar. The *P*-values for comparison between two groups were obtained by Student’s *t*-test (two-tailed). The Welch’s correction of *t*-test was performed when the variances in two groups were unequal. All values were expressed as mean±s.d. and the *P-*value of <0.05 was considered to be statistically significant.

## Publisher’s note

Springer Nature remains neutral with regard to jurisdictional claims in published maps and institutional affiliations.
