# Supplementary figures and images for: Impaired p65 degradation by decreased chaperone-mediated autophagy activity facilitates epithelial-to-mesenchymal transition
Source: Oncogenesis. 2017 Oct 9;6(10):e387–. doi: 10.1038/oncsis.2017.85 (PMC5668883; doi:10.1038/oncsis.2017.85)

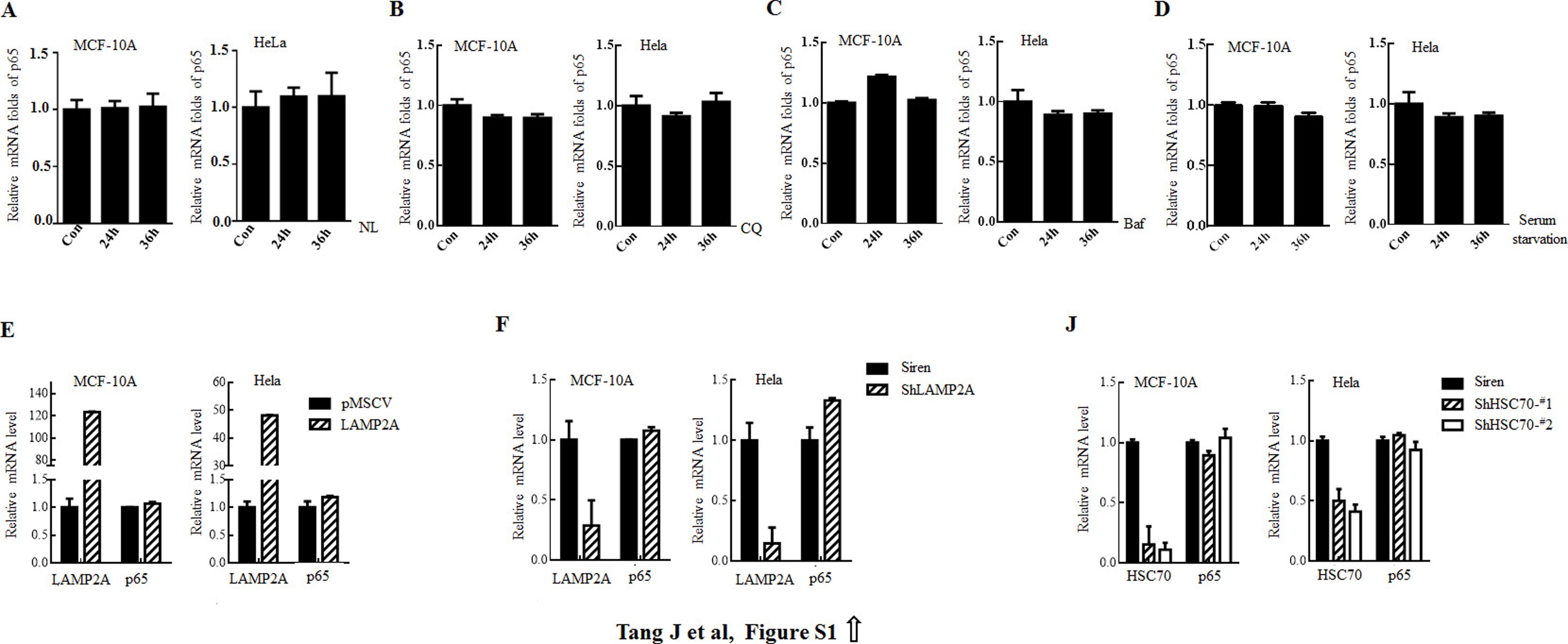

Supplement: Supplementary Figure 1 [file oncsis201785x1.tif]

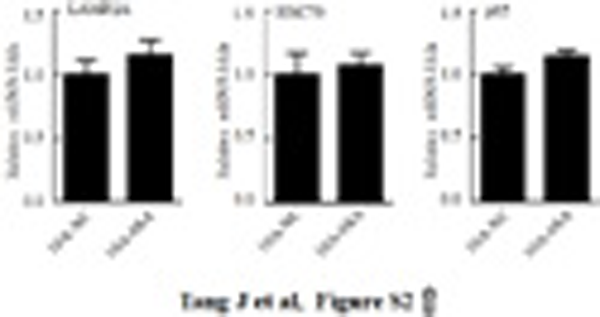

Supplement: Supplementary Figure 2 [file oncsis201785x2.tif]

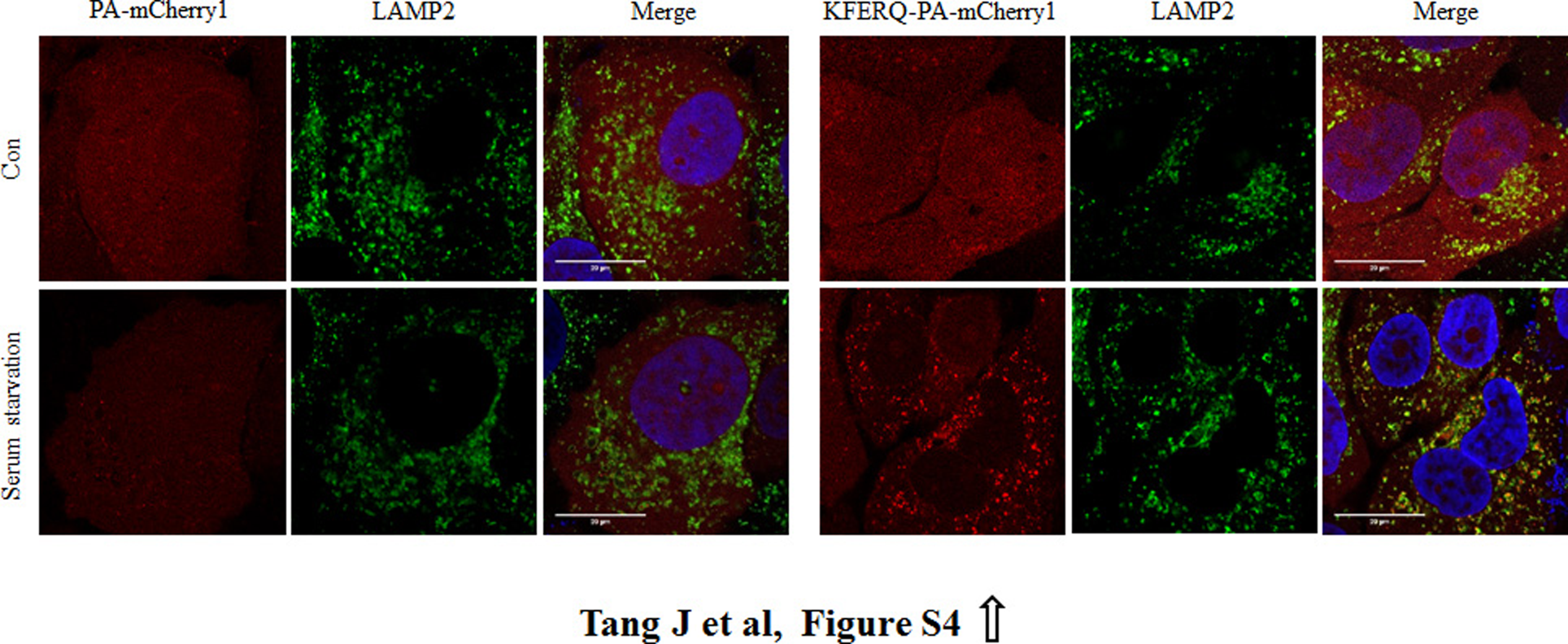

Supplement: Supplementary Figure 4 [file oncsis201785x4.tif]
