# Supplementary material for: Impaired p65 degradation by decreased chaperone-mediated autophagy activity facilitates epithelial-to-mesenchymal transition
Source: Oncogenesis. 2017 Oct 9;6(10):e387–. doi: 10.1038/oncsis.2017.85 (PMC5668883; doi:10.1038/oncsis.2017.85)
Supplement: Supplementary Figure Legends [file oncsis201785x5.docx]

**Figure S1**

(A-D). MCF-10A and Hela cells were exposed to treatment with serum starvation, CQ (100 μM), Baf (10 nM), and NL(20 mM NH_4_Cl and 100 μM leupeptin) for indicated time respectively, and the transcription level of p65 was detected by quantitative real-time PCR. (E-G). MCF-10A and HeLa cells were stably infected with LAMP2A overexpression vector (E), shRNA against LAMP2A (F) and shRNA against HSC70 (G) respectively, followed by quantitative real-time PCR for detection of indicated genes.

**Figure S2**.

The folds of relative mRNA levels of LAMP2A, HSC70 and p65 against MCF-10A-NC cells were quantified by quantitative real-time PCR in MCF-10A-6SA cells.

**Figure S3.**

(A-D) MCF-10A-NC and MCF-10A-6SA cells were treated with MG132 (20 μM)，6-AN (100 μM)， bafilomycin (10 nM)，and CQ (50 μM) for indicated time respectively, and cell lysates were analyzed for indicated proteins with β-actin used as the internal control (left) . Right. Quantification analysis of the band density of p65 as analyzed by Image J and normalized to that of β-actin.

**Figure S4.**

MCF-10A cells were infected with PA-mCherry1 or KFERQ-PA-mCherry1, and after photoactivation by 405-nm light they were maintained in medium with or without serum for 20-h. Immunofluorescence co-staining of LAMP2 and DAPI in these cells. Scale bar represents 20 μm.
