# Supplementary material for: Impaired p65 degradation by decreased chaperone-mediated autophagy activity facilitates epithelial-to-mesenchymal transition
Source: Oncogenesis. 2017 Oct 9;6(10):e387–. doi: 10.1038/oncsis.2017.85 (PMC5668883; doi:10.1038/oncsis.2017.85)
Supplement: Supplementary Table 1 [file oncsis201785x6.docx]

**Supplementary table 1. Targeting sequences of shRNAs against indicated genes**

| shRNA | Sequence 5’→3’ |
| --- | --- |
| shHSC70-^#^1 | AGTTTAAGCGCAAGCATAA |
| shHSC70-^#^2 | GAACAAGAGAGCTGTAAGA |
| shLAMP2A | TGAACATCACTCAGGATAA |
| shATG7-^#^1 | GCTTTGGGATTTGACACATTT |
| shATG7-^#^2 | GGCGTGAGACACATCACATTT |
| shp65-^#^1 | GATGAGATCTTCCTACTGT |
| shp65-^#^2 | GGATTGAGGAGAAACGTAA |
