# Supplementary material for: Impaired p65 degradation by decreased chaperone-mediated autophagy activity facilitates epithelial-to-mesenchymal transition
Source: Oncogenesis. 2017 Oct 9;6(10):e387–. doi: 10.1038/oncsis.2017.85 (PMC5668883; doi:10.1038/oncsis.2017.85)
Supplement: Supplementary Table 2 [file oncsis201785x7.docx]

**Supplementary Table 2. Real time PCR primer sequences for indicated genes**

| Gene | Forward primer 5’→3’ | Reverse Primer 5’→3’ |
| --- | --- | --- |
| *p65* | ATGTGGAGATCATTGAGCAGC | CCTGGTCCTGTGTAGCCATT |
| *HSC70* | ACCTACTCTTGTGTGGGTGTT | GACATAGCTTGGAGTGGTTCG |
| *LAMP2A* | TGCAGTGCAGATGACGAC | GTGCTTGAGACCAATAAAATAA |
