# Supplementary material for: Impaired p65 degradation by decreased chaperone-mediated autophagy activity facilitates epithelial-to-mesenchymal transition
Source: Oncogenesis. 2017 Oct 9;6(10):e387–. doi: 10.1038/oncsis.2017.85 (PMC5668883; doi:10.1038/oncsis.2017.85)
Supplement: Supplementary Table 3 [file oncsis201785x8.docx]

**Supplementary Table 3. Primer pairs for site directed mutagenesis**

| Primer | Sequence 5’→3’ |
| --- | --- |
| Q119AA-F | CTGGGAATCGCG GCT GTGAAGAAGCGGGACCTGGAGCAG |
| Q119AA-R | CTTCTTCACAGCCGCGATTCCCAGGTTCTGGAAACTGTG |
| Q162AA-F | CGGCTCTGCGCC GCG GTGACAGTGCGGGACCCATCAGGC |
| Q162AA-R | CACTGTCACCGCGGCGCAGAGCCGCACAGCATTCAGGTC |
| Q220AA-F | GACAAGGTGGCG GCA GAGGACATTGAGGTGTATTTCACG |
| Q220AA-R | AATGTCCTCTGCCGCCACCTTGTCACACAGTAGGAAGAT |
| Q247AA-F | GATGTGCACGCA GCA GTGGCCATTGTGTTCCGGACCCCT |
| Q247AA-R | AATGGCCACTGCTGCGTGCACATCAGCTTGCGAAAAGGA |
