# Supplementary material for: Impaired p65 degradation by decreased chaperone-mediated autophagy activity facilitates epithelial-to-mesenchymal transition
Source: Oncogenesis. 2017 Oct 9;6(10):e387–. doi: 10.1038/oncsis.2017.85 (PMC5668883; doi:10.1038/oncsis.2017.85)
Supplement: Supplementary Figure [file oncsis201785x9.docx]

**Figure S4. Antibodies used in this study**

| Antibody | Company | Catalog number |
| --- | --- | --- |
| β-catenin | Cell Signaling Technology | #9587 |
| E-cadherin | Cell Signaling Technology | #4065 |
| Snail | Cell Signaling Technology | #3879 |
| Vimentin | Cell Signaling Technology | #5742 |
| PKM2 | Cell Signaling Technology | #4053 |
| p65 | Santa Cruz | sc372 |
| HSC70 | Santa Cruz | sc7298 |
| Lamp2 | Santa Cruz | sc18822 |
| P53 | Santa Cruz | sc-6243 |
| IκBα | Santa Cruz | sc847 |
| p65 | Abcam | ab32536 |
| LAMP2A | Abcam | ab125068 |
| LRRK2 | Abcam | ab133518 |
| ATG7 | Abcam | ab133528 |
| GAPDH | Abmart | M20006 |
| GST | Abmart | M20007 |
| β- actin HRP-DirecT | MBL | PM053-7 |
| P62 | MBL | PM045B |
| Fibronectin | BD Biosciences | 610078 |
| His | Beyotime | AH367 |
